# Diminazene Aceturate (Berenil) Modulates the Host Cellular and Inflammatory Responses to *Trypanosoma congolense* Infection

**DOI:** 10.1371/journal.pone.0048696

**Published:** 2012-11-07

**Authors:** Shiby Kuriakose, Helen M. Muleme, Chukwunonso Onyilagha, Rani Singh, Ping Jia, Jude E. Uzonna

**Affiliations:** Department of Immunology, University of Manitoba, Winnipeg, Manitoba, Canada; Karolinska Institutet, Sweden

## Abstract

**Background:**

*Trypanosoma congolense* are extracellular and intravascular blood parasites that cause debilitating acute or chronic disease in cattle and other domestic animals. Diminazene aceturate (Berenil) has been widely used as a chemotherapeutic agent for trypanosomiasis in livestock since 1955. As in livestock, treatment of infected highly susceptible BALB/c mice with Berenil leads to rapid control of parasitemia and survival from an otherwise lethal infection. The molecular and biochemical mechanisms of action of Berenil are still not very well defined and its effect on the host immune system has remained relatively unstudied. Here, we investigated whether Berenil has, in addition to its trypanolytic effect, a modulatory effect on the host immune response to *Trypanosoma congolense*.

**Methodology/Principal Findings:**

BALB/c and C57BL/6 mice were infected intraperitoneally with *T. congolense*, treated with Berenil and the expression of CD25 and FoxP3 on splenic cells was assessed directly ex vivo. In addition, serum levels and spontaneous and LPS-induced production of pro-inflammatory cytokines by splenic and hepatic CD11b^+^ cells were determined by ELISA. Berenil treatment significantly reduced the percentages of CD25^+^ cells, a concomitant reduction in the percentage of regulatory (CD4^+^Foxp3^+^) T cells and a striking reduction in serum levels of disease exacerbating pro-inflammatory cytokines including IL-6, IL-12, TNF and IFN-γ. Furthermore, Berenil treatment significantly suppressed spontaneous and LPS-induced production of inflammatory cytokines by splenic and liver macrophages and significantly ameliorated LPS-induced septic shock and the associated cytokine storm.

**Conclusions/Significance:**

Collectively, these results provide evidence that in addition to its direct trypanolytic effect, Berenil also modulates the host immune response to the parasite in a manner that dampen excessive immune activation and production of pathology-promoting pro-inflammatory cytokines, suggesting that this drug may also be beneficial for treatment of disease conditions caused by excessive production of inflammatory cytokines.

## Introduction

African trypanosomiases are diseases of humans and livestock caused by several species of flagellated single-celled protozoan parasites belonging to the genus *Trypanosoma*. Trypanosomes are transmitted from infected to uninfected animals by different species of tsetse fly during regular blood meals. They remain in the bloodstream as extracellular parasites and therefore are constantly exposed to the host’s immune system. As a result, African trypanosomes have developed sophisticated immune evasion mechanisms including antigenic variation [1], [2], excessive activation of the complement system leading to hypocomplementemia [2], [3], polyclonal B cell activation [4]and immunosuppression [5].

Trypanosomiasis in animals is caused by *Trypanosoma congolense*, *Trypanosoma brucei brucei* and *Trypanosoma vivax*, and is much the same disease in livestock as it is in humans. The cattle industry in many African countries is hit particularly hard; it is estimated that the disease costs $1.3 billion to livestock producers and consumers every year [6]. Of the three species of animal trypanosomiasis, *T. congolense* is the most important disease for livestock [7]. While other species, particularly *T. brucei*, has the capacity to invade the capillary interstitial walls, *T. congolense* are purely intravascular and hence unable to leave the circulation [7]. Thus, they are continuously exposed and interact with the host circulatory defense factors leading to extreme immunopathology.

BALB/c mice are highly susceptible to experimental *T. congolense* infection and succumb to the infection within 8–10 days [8]. Death of infected BALB/c mice is usually related to immune hyper-activation of cells, particularly macrophages and T cells, leading to massive production of pro-inflammatory cytokines (including IFN-γ, IL-1, IL-6, IL-12 and TNF-α and systemic inflammatory response syndrome (SIRS) [9] In contrast, C57BL/6 mice are considered relatively resistant to *T. congolense* infection because they can control several waves of parasitemia and survive for over 100 days [9]. These mice produce low levels of pathology-inducing pro-inflammatory cytokines and their immune cells are relatively quiescent or hypo-activated [9]. Complement and antibody-mediated phagocytosis by splenic and liver (Kupffer cells) macrophages is one of the primary mechanism by which trypanosomes are cleared from an infected host [10]. However, these cells also contribute to the excessive production of pro-inflammatory cytokines following their interaction with the parasites [10].

Chemotherapeutic agents used for treatment of animal trypanosomiasis include suramin, dimiazene aceturate (Berenil), isometamidium and homidium. Berenil has been in use as an anti-trypanosome drug for livestock since 1955. The main biochemical mechanism of Berenil’s trypanocidal actions is thought to be by binding to kinetoplast DNA [11] thereby inducing complete and irreversible loss of kDNA in certain strains of trypanosomes [12], [13]. Due to its molecular structure, Berenil has particular affinity for A-T base pairs in circular DNA and kinetoplast DNA [11], [14], [15]. Berenil is not licensed for use in humans because of serious side-effects observed in animals, which include tremors, itching, sweating, convulsions, dyspnea, recumbency and vomiting in camels [13] and decreased blood pressure [16]and diarrhea in dogs.

Despite its use for over 50 years, few studies have investigated the ability of Berenil to modulate the host immune responses. Plasma from Berenil-treated cattle showed significant *in vitro* anti-trypanosome activity for up to 3 weeks after a single intramuscular injection, and mice treated with Berenil before infection are protected against homologous challenge up to 42 days post treatment [17]. It has been shown that treatment of *T. congolense*-infected BALB/c mice with Berenil alters the nature of their B cell (antibody) responses, increasing protective IgG2a and IgG3 responses against VSG and whole parasite [18]. In addition, Berenil treatment also abolishes *T. congolense*-induced immunosuppression *in vitro* and *in vivo*, allowing the animals to mount successful immune responses against secondary challenge with a different pathogen [19]. Tabel and Otesile [20] showed that BALB/c mice cured of *T. congolense* infection with Berenil and challenged with a homologous strain of the parasite could control infection for up to 36 days post challenge. This response could not be achieved with either serum transfer from infected mice alone or with Berenil treatment of naïve (uninfected) mice. Furthermore, parasites isolated from BALB/c mice after challenge were found to be a different variant from the injected strain and mice could not control challenge with a heterologous strain. Taken together, these studies indirectly suggest that Berenil administered during infection modulates the host immune response.

In this paper, we have examined several immune parameters of mice infected with *T. congolense* and treated with Berenil to investigate whether the drug modulates the host immune response to the parasite. We show that Berenil treatment reduced serum levels of pro-inflammatory cytokines, and alters the activation status of lymphocytes in the spleens and livers of infected mice. These effects could augment the trypanolytic activities of the compound leading to more effective parasite and disease control.

## Materials and Methods

### Mice

Six to eight week old female BALB/c, C57BL/6 and outbred Swiss white (CD1) mice were purchased from Central Animal Care Services, University of Manitoba (Winnipeg, Canada). All mouse experiments were approved by the University of Manitoba Animal Care Committee in accordance with the regulation of the Canadian Council on Animal Care.

### Parasite

*Trypanosoma congolense*, Trans Mara strain, variant antigenic type (TC13) was used in this study. The origin of this parasite strain has been described previously [21]. Frozen stabilates of parasites were used to infect CD1 mice previously immunosuppressed (48 hr prior to infection) with cyclophosphamide (0.2 mg/kg). Infected CD1 mice were sacrificed after 3 days and parasites were purified from their blood by DEAE-cellulose chromatography [22], washed in Tris-saline buffer containing 5% glucose and 10% heat-inactivated fetal bovine serum and used to infect BALB/c mice and C57BL/6 mice.

### Infection and Estimation of Parasitemia

For infection, groups of mice were injected i.p. with 10^3^ *T. congolense* variant antigenic type TC13. At 5 days post-infection, some mice were treated i.p. with diminazene aceturate (Berenil, 14 mg/kg, Sigma Aldrich, St. Louis MO) in PBS and the controls were injected with PBS. For estimation of parasitemia, a drop of blood was taken from the tail vein of each infected mouse, mounted on a microscopic slide and covered with a cover slip. Parasitemia was estimated by counting the number of parasites present in 3–5 fields at 400x magnification by light microscopy.

### Isolation of Spleen Cells, Culture and ex vivo Regulatory T cell Staining

At various times after infection, mice were sacrificed and the spleens were harvested and made into single cell suspensions. Cells were washed and red blood cells were lysed with ACK lysis buffer. The cells were counted, resuspended at 4 million/ml in complete medium (DMEM supplemented with 10% heat-inactivated FBS, 2 mM L-glutamine, 100 U/ml penicillin, and 100 µg/ml streptomycin), plated at 1ml/well in 24-well tissue culture plates (Falcon, VWR Edmonton, Canada) and cultured at 37°C in a CO_2_ incubator. After 72 hr, the culture supernatant fluids were collected and stored at –20°C until assayed for cytokines by ELISA. In some experiments, splenic CD11b^+^ cells were isolated by positive selection using AUTOMACS column and antibodies from Miltenyi (Miltenyi Biotec Inc, Auburn, CA) according to the manufacturer’s suggested protocol.

### Isolation of Liver Macrophages (Kupffer Cells)

To isolate kupffer cells, infected or uninfected mice were anesthetized with isoflourane and blood was collected by cardiac puncture. The chest cavity was opened and the livers were perfused by injecting 10 ml ice-cold PBS into the right ventricle. Thereafter, the liver were minced in collagenase solution (1mg/ml), digested at 37°C for 1 hour and passed through a 70 µm cell strainer (VWR, ON, Canada). Cells were washed with 30 ml Hanks balanced salt solution (HBSS) (Invitrogen, ON, Canada) at 1200 rpm for 5 min. Contaminating red blood cells were lysed with ACK lysis buffer, washed once with HBSS and the cells were resuspended in 4 ml 40% percoll (Sigma). Liver lymphocytes were separated by layering the cells on top of 70% percoll (Sigma) and centrifuging at 750 g at 22°C for 20 min without brakes. The interface containing the mononuclear cells was carefully collected, washed twice with PBS and re-suspended in complete DMEM medium. CD11b^+^ cells were then enriched by positive selection using AUTOMACS (Miltenyi Biotec). Enriched liver CD11b^+^ cells were greater than 96% positive for F4/80 expression as assessed by flow cytometry. The cells were washed, counted and cultured for 24 hr in the presence or absence of LPS (1 µg/mg) and culture supernatant fluids were assayed for IL-6, TNF and IL-12 by ELISA.

### Flow Cytometry

At sacrifice, single cell suspensions were made from spleens of *T. congolense*-infected mice and stained directly *ex vivo*. Intracellular staining for Foxp3 was performed using the Treg Staining Kit (eBioscience) in accordance with the manufacturer’s recommendations. In brief, cells were treated with fixative/permeabilization buffer, washed and intracellular staining was then performed using PE-conjugated anti-Foxp3 antibody, APC-conjugated anti-CD25 and FITC-conjugated CD4. Samples were resuspended in FACS staining buffer and analyzed on a BD FACS Canto II flow cytometer using Diva software (BD Biosciences).

### Induction of Septic Shock

To determine the influence of Berenil on LPS-induced septic shock, BALB/c mice were injected with Berenil 24 hr prior to being challenged intraperitoneally with LPS (5 mg/kg). Mice were monitored for movement, body condition and alertness every 3 hr and disease severity was scored in a semi-quantitative fashion as follows: 0, = no abnormal clinical sign; 1, = ruffled fur but lively; 2, = ruffled fur, moving slowly, hunched, and sick; 3, = ruffled fur, squeezed eye, hardly moving, down and very sick; 4, = moribund; and 5, = dead. Clinical score 4 was used as the humane endpoint because the institutional ethical regulation does not permit score 5 in all animal experiments. Mice were sacrificed after 24 hr and peritoneal wash fluid and serum were collected for cytokine analysis.

### Serum Cytokine Analysis

At sacrifice, blood samples were taken by cardiac puncture and serum was separated by centrifugation and stored at −20°C until assayed for cytokines. Serum levels of IL-6, IL-12p40, TNF-α and IFN-γ were determined by sandwich ELISA using antibody pairs purchased from Biolegend (San Diego, CA). The sensitivity of the ELISA ranges between 7.5–15 pg/ml for all analytes.

### Statistical Analysis

A two-tailed Student’s t-test was used to compare data means from different groups of mice. Data are presented as means ± SE. Significance was considered if p<0.05. All analyses were carried out using GraphPad Prism software.

## Results

### Treatment with Berenil Prevents Early Death of Infected BALB/c Mice and Alters the Activation Status of Lymphocytes and Frequency of Regulatory T cells in Spleens of Infected Mice

BALB/c mice infected with 10^3^ *Trypanosoma congolense* are unable to control their first wave of parasitemia and die acutely with mean survival time of 8±1 day (data not shown),[18]. Treatment of infected mice with Berenil (14 mg/kg i.p.) on day 5 post-infection led to clearance of parasitemia by day 7 post-infection and an indefinite survival (data not shown).

**Figure 1:**
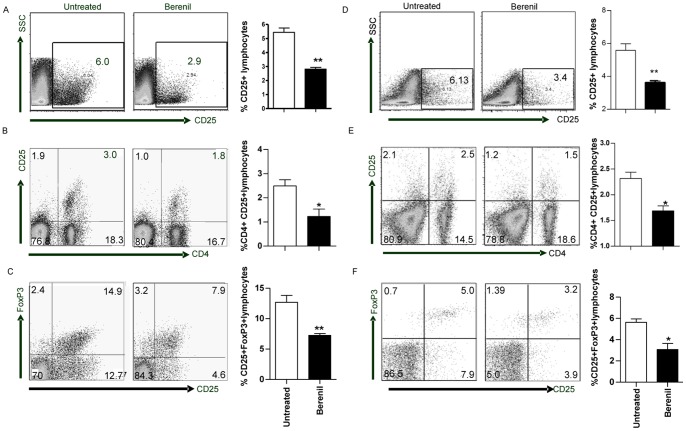
Berenil treatment decreases the percentage of CD25^+^ and FoxP3^+^ cells in the spleens of infected mice. Splenocytes from *Trypanosoma congolense*-infected BALB/c and C57BL/6 mice either treated or untreated with Berenil were routinely stained (see materials and methods) directly *ex vivo* with fluorochrome-conjugated mAb against CD4, CD25 and Foxp3 and analyzed by flow cytometry. Shown are representative dot plots showing expression of CD25 on total (A and D) and CD4^+^ (B and E) lymphocytes. Representative dot plot of CD25^+^ and Foxp3^+^ cells gated on CD4^+^ lymphocytes (C and F). The bar graphs represent the cumulative percentages of CD25^+^ and Foxp3^+^ cells from 3–5 mice per group. The results presented are representative of 4 different experiments with similar results. Bars show mean +/−SEM; *, p<0.05; **, p<0.01.

The susceptibility of BALB/c mice to *T. congolense* has been associated with immune cell hyper-activation particularly T cells and macrophages [23]. Furthermore, recent reports suggest that regulatory T cells play important roles in the pathogenesis of *T. congolense* infection in mice [5], [23], [24] Therefore, we investigated the effects of Berenil treatment on CD25 expression (an activation marker) on lymphocytes and FoxP3 expression (regulatory T cell marker) on CD4^+^ T cells from spleens of treated and untreated mice. There was a marked (50%) reduction in CD25 expression on total lymphocytes from Berenil-treated Balb/c mice (**Figure. 1A**). The reduction in the percentage of CD4^+^CD25^+^ cells though significant was not as pronounced as that seen for total lymphocytes (**Figure. 1B**), suggesting that most of this change was in another lymphocyte population. Furthermore, Berenil-treated Balb/c mice had significantly lower numbers of CD4^+^CD25^+^FoxP3^+^ expressing cells than untreated mice (**Figure. 1C**). Similar results were also obtained in infected and treated relatively resistant C57BL/6 mice **(** **Figure. 1D–F**). Interestingly, Berenil treatment did not alter the frequency of CD4^+^CD25^+^FoxP3^+^ cells in the spleens of uninfected mice (Figure S1), suggesting that the effect observed in infected mice may be related to changes in the dynamics of Tregs due to *T. congolense* infection [5], [23], [24].

### Berenil Treatment Reduces Systemic Levels of Pro-inflammatory Cytokines in *T. congolense-*infected Mice

**Figure 2:**
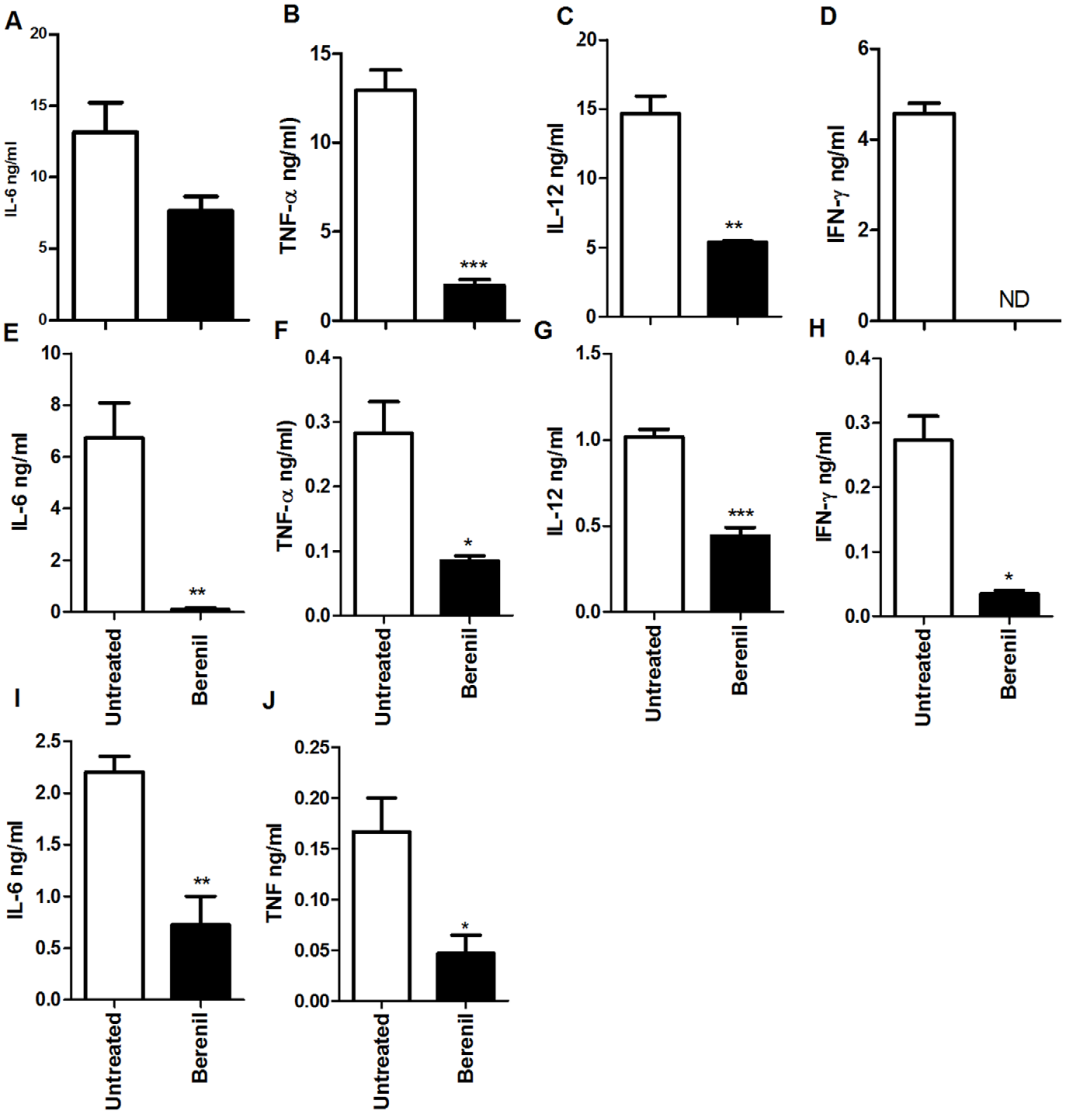
Treatment with Berenil reduces serum pro-inflammatory cytokine levels in *T. congolense*-infected mice. BALB/c (A–D) and C57BL/6 (E–H) mice were infected with *T. congolense* and treated with Berenil on day 5 post-infection. Eight days post-infection (3 days after Berenil treatment), mice were sacrificed and blood samples were taken via cardiac puncture to obtain serum. The levels of IL-6 (A and E), TNF (B and F), IL-12 (C and G) and IFN-γ (D and H) in the serum were determined by sandwich ELISA. Uninfected BALB/c mice were also treated with Berenil, sacrificed 3 days later and serum levels of IL-6 (I) and TNF (J) were determined by ELISA. The results presented are representative of 3 (A–H) and 2 (I and J) independent experiments (n = 4 mice per group) with similar results. Bars show mean +/−SEM; *, p<0.05; **, p<0.01; ***, p<0.001. N.D. = not detected (i.e. below 15 pg/ml).

Acute death of *T. congolense*-infected BALB/c and IL-10R deficient C57BL/6 mice is usually attributed to excessive production of inflammatory cytokines by immune cells leading to a cytokine storm and concomitant systemic inflammatory response syndrome [5]. Therefore, we also determined whether Berenil treatment was also associated with reduction in the production of inflammatory cytokines. The levels of several pro-inflammatory cytokines (IL-6, TNF, IL-12, and IFN-γ) were significantly reduced (by several fold) in Berenil-treated BALB/c mice (**Figure. 2A–D**). This reduction was most dramatic for IFN-γ, which was below detectable levels in the treated group (**Figure. 2D**). Similar results were also obtained in infected and treated C57BL/6 mice (**Figure. 2E–H**), suggesting that the effect of Berenil is not mouse strain specific. Paradoxically, Berenil treatment also caused a significant reduction in serum levels of IL-10 in infected BALB/c mice (348±46 pg/ml vs. 56±23 pg/ml, p<0.03 for untreated vs. treated groups, respectively). Interestingly, Berenil treatment of naïve (uninfected) BALB/c mice also caused significant reduction in serum levels of IL-6 (**Figure. 2I**) and TNF-α, (**Figure. 2J**) although IL-12 and IFN-γ were below detectable levels. These results suggest that the reduction in serum levels of pro-inflammatory cytokines in *T. congolense*-infected mice was not solely due to destruction of parasites and subsequent reduction in parasitemia by Berenil.

### Berenil Treatment Alters the Responsiveness of Splenic and Hepatic CD11b^+^ cells to LPS Stimulation

**Figure 3:**
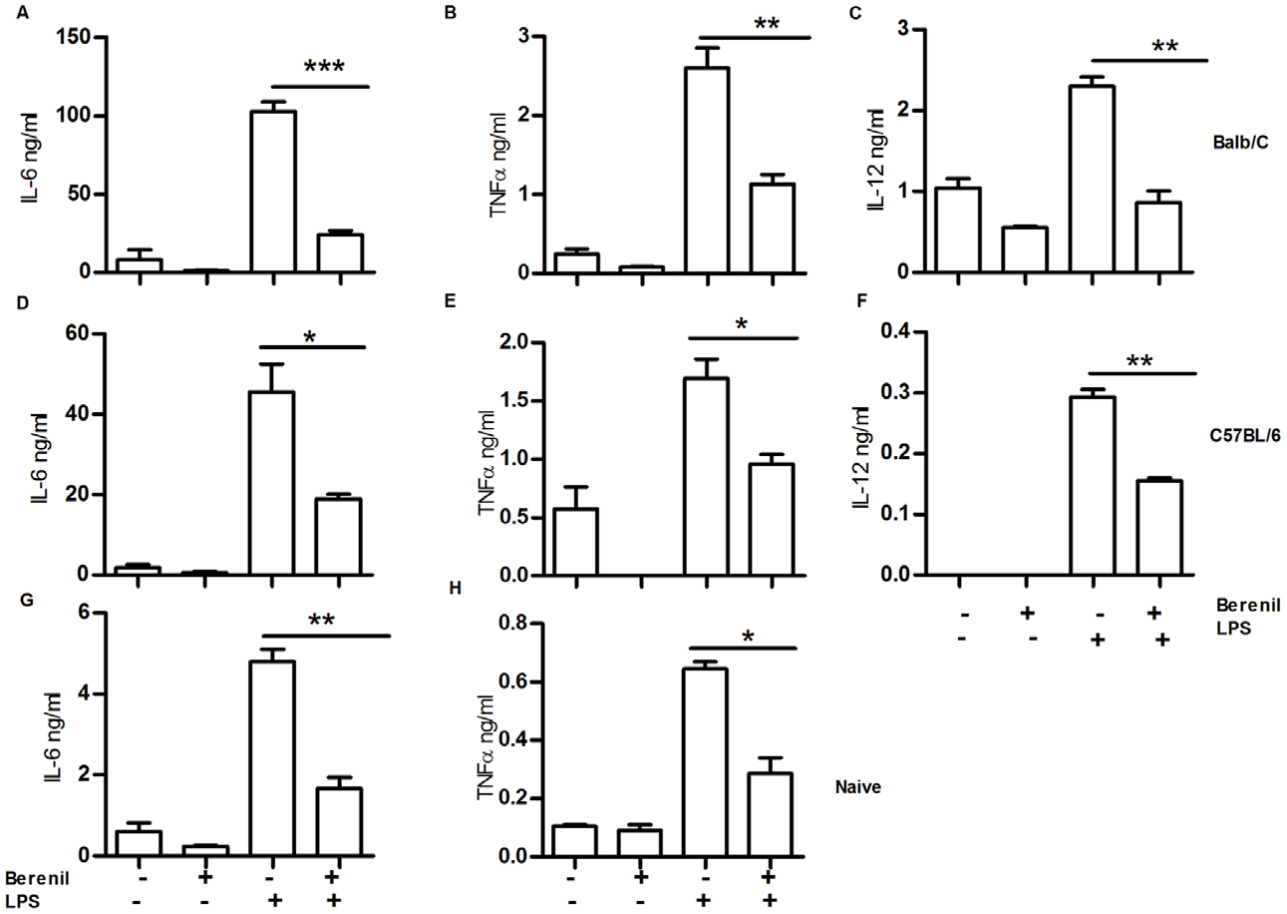
Berenil treatment suppresses IL-6, IL-12 and TNF production by CD11b^+^ spleen cells from *T. congolense* infected mice. Highly enriched (by positive selection) CD11b^+^ splenocytes from Berenil treated or non-treated infected (A–F) and uninfected (G and H) mice were cultured for 18 hours with or without LPS (5µg/ml) and the culture supernatant fluids were assayed for IL-6 (A, D and G), TNF (B, E and H) and IL-12p40 (C and F) by ELISA. Top (A, B and C) and middle (D, E and F) panels are data obtained with splenocytes from infected BALB/c and C57BL6 mice, respectively. The bottom panel (G and H) shows data from uninfected (naïve) C57BL/6 mice. The results presented are representative of 3 independent experiments (n = 4 mice per group) with similar results. Bars show mean +/−SEM; *, p<0.05; **, p<0.01; ***, p<0.001. N.D. = not detected (i.e. below 15 pg/ml).

Because of their role in phagocytosis and production of inflammatory cytokines, CD11b^+^ cells, particularly macrophages are vital for both clearance of *T. congolense* and in mediating immunopathology in infected mice [10]. Therefore, we examined the influence of Berenil on *ex vivo* purified CD11b^+^ cells from spleens of *T. congolense* infected or uninfected mice following *in vitro* stimulation with or without LPS (5 µg/ml). Interestingly, the absolute numbers of CD11b^+^ cells from spleens of Berenil-treated mice were significantly higher than those from untreated mice (106.3±14.3 vs. 66.7±5.5, p = 0.011 for Berenil-treated and untreated animals, respectively). This suggests that alteration in numbers of CD11b^+^ cells is not responsible for the reduction in serum levels of pro-inflammatory cytokines observed in Berenil-treated mice (Figures 2A–H). Berenil treatment significantly (p<0.05) suppressed spontaneous as well as LPS-induced production of IL-6, TNF and IL-12p40 by splenic CD11b^+^ cells from infected BALB/c and C57BL/6 mice (**Figure. 3A–F**). Similar results were also obtained from CD11b^+^ cells from uninfected mice (Figure. 3G and H), although IL-12 production by cells from uninfected mice was below detectable levels.

### Berenil Treatment Reduces Pro-inflammatory Cytokine Secretion by Kupffer Cells from *T. congolense* Infected Mice

**Figure 4:**
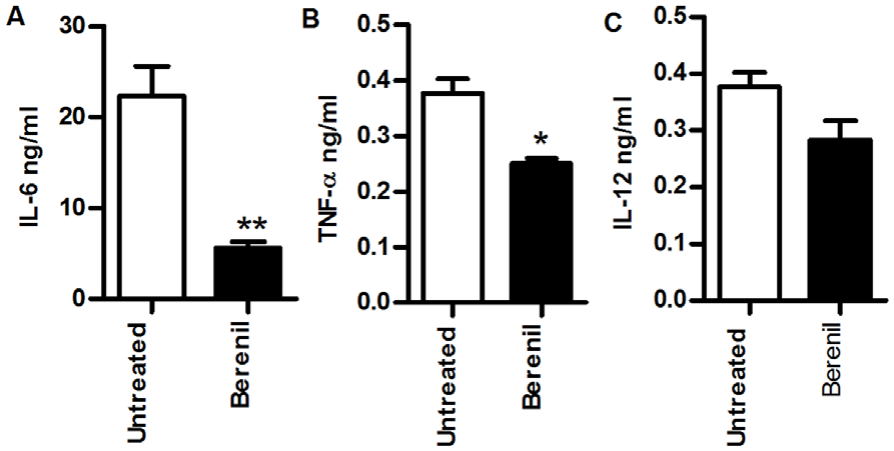
Berenil treatment reduces spontaneous pro-inflammatory cytokine secretion by kupffer cells from *T. congolense*-infected mice. BALB/c mice were infected with *T. congolense* and treated with Berenil on day 5 post-infection. Eight days post-infection (3 days after Berenil treatment), mice were sacrificed and hepatic mononuclear cells were isolated by Percoll gradient centrifugation. Kupffer cells were further enriched by positive selection using CD11b-coated beads, cultured for 24 hr and the concentration of IL-6 (A), TNF (B) and IL-12 (C) in culture supernatant fluids were measured by ELISA. The results presented are representative of 2 independent experiments (n = 4 mice per group) with similar results. Bars show mean +/−SEM; *, p<0.05; **, p<0.01.

Because clearance of trypanosomes in infected mice is mediated primarily by liver macrophages [10], we isolated kupffer cells from livers of infected mice at Day 8 post-infection and assessed their production of cytokines directly *ex vivo*. We chose to look at Day 8 post-infection because previous report has shown this time to be the peak of kupffer cell activity and maximum parasite uptake after *T. congolense* infection [10]. As shown in **Figure 4A and B**, Berenil treatment significantly reduced the spontaneous (directly *ex vivo*) IL-6 and TNF production by kupffer cells from infected mice. Similar effects were also seen for IL-12p40 production although this was not statistically significant (**Figure. 4C**). Taken together, these findings suggest that Berenil can specifically alter macrophage responses *in vivo* by reducing their ability to respond to certain pathogen-derived stimuli.

### Berenil Ameliorates LPS-induced Systemic Inflammatory Response Syndrome

**Figure 5:**
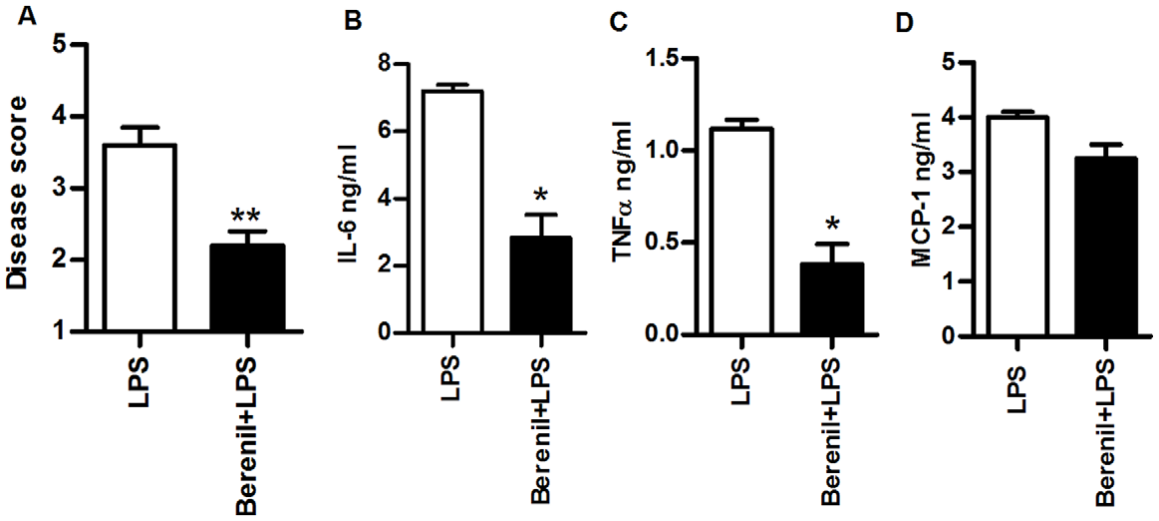
Berenil ameliorates LPS-induced toxicity and production of pro-inflammatory cytokines *in vivo*. Naïve BALB/c mice were injected with Berenil and after 24 hr challenged with LPS (10 µg/ml) intraperitoneally. After 24 hr, mice were assessed for clinical (disease) score (A), sacrificed and serum levels of IL-6 (B), TNF (C), and MCP-1 (D) were determined by ELISA. The results presented are representative of 2 independent experiments (n = 4 mice per group) with similar results. Bars show mean +/−SEM; *, p<0.05.

The preceding results suggest that Berenil might also alter the overall host inflammatory response to microbial stimuli. Therefore, we assessed the effects of Berenil on LPS-induced model of septic shock. As shown in **Figure 5A–D**, pretreatment of mice with Berenil significantly ameliorated LPS-induced toxicity as evidenced by reduction in the overall disease score (Figure. 5A) and significant reduction in serum levels of IL-6 (Figure. 5B) and TNF (Figure. 5C). Berenil treatment also lowers serum levels of MCP-1 although this was not statistically significant (Figure. 5D). Collectively, these findings show that Berenil dampens systemic inflammatory response by altering responsiveness of immune cells to microbial stimuli.

## Discussion

The primary objective of this study was to determine whether diminazine aceturate (Berenil) has, in addition to its trypanolytic property, an immunodulatory effect following infection with *Trypanosoma congolense*. Acute death of *T. congolense-*infected susceptible (BALB/c) mice has been attributed to immune hyper-activation (particularly of macrophages and T cells), which leads to concomitant over production of pro-inflammatory cytokines, SIRS, immunopathology and death [5]. In addition, regulatory T cells have been implicated in enhanced susceptibility to experimental *T. congolense* infection [23]. In the present study, we showed that Berenil treatment significantly lowers the serum levels of pro-inflammatory cytokines in both the highly susceptible and relatively resistant mice. Furthermore, we showed that Berenil treatment dampens immune cell activation as evidenced by significantly lower percentages of CD25 expressing lymphocytes in spleens of infected mice. Collectively, these observations suggest that survival of *T. congolense*-infected highly susceptible BALB/c mice following Berenil treatment may be related in part to the dampening of immune activation and pro-inflammatory cytokine production.

In addition to lower immune activation, we also found that the percentage of CD4^+^Foxp3^+^ (T reg) cells was significantly lower in infected mice after Berenil treatment (**Figure. 1C and F**). We speculate that the lower systemic inflammatory response in Berenil-treated mice may result in a lower necessity for Tregs leading to their impaired proliferation via unknown feedback mechanisms. The role of Tregs in experimental *T. congolense* infection is controversial. They have been shown to prevent effective control of parasitemia such that when BALB/c mice were treated with low doses of anti-CD25 depleting antibody (PC61), they became resistant to *T. congolense* infection [23]. In contrast, another study found that IL-10-producing FoxP3^+^ T regs are required for decreasing excessive macrophage activation [24]. We recently found that treatment of mice with PC61 prior to infection leads to an increased prepatent period and reduced peak parasitemia in both the highly susceptible and relatively resistant mice [25]. In fact, depletion of Tregs by a single treatment of anti-CD25 mAb prior to infection resulted in clearance of first wave of parasitemia and significantly increased the survival period in the highly susceptible BALB/c mice that normally do not control first wave of parasitemia [25].Thus, it is conceivable that the enhanced resistance in mice following treatment with Berenil may in part be related to its effect in lowering Tregs in infected mice. Interestingly, Berenil treatment did not alter the frequency of Tregs in naïve mice, contrary to the observation in *T. congolense*-infected mice. This suggests that the reduction in Treg numbers observed in infected and treated mice might be related to infection-induced increase in numbers of these cells. However, Berenil treatment caused a significant reduction in serum levels of IL-6 and TNF in naïve animals (see **Fig. 2I** and J). Collectively, these observations suggest that the downregulatory effect of Berenil on pro-inflammatory cytokine production is distinct and not related to its effect on Tregs.

We found that Berenil treatment also caused significant reduction in serum IL-10 levels in infected mice. Although IL-10 has a well-documented anti-inflammatory role in *T. congolense* infection, it has also been shown to mediate trypanosome-induced immunosuppression [8], [9], [26], [27]. Previous work has shown that following Berenil treatment of infected mice, immunosuppression is alleviated and mice were able to successfully respond to secondary bacterial infection [19]. It is possible that in addition to clearing parasites, Berenil contributes to restoring immune responses by decreasing systemic overproduction of IL-10. Alternatively, it is possible that Berenil has a global suppressive effect on cytokine release by activated macrophages. In addition to IL-6, TNF-α and IL-12, macrophages produce copious amounts of IL-10 following their interaction with trypanosomal antigens [27]. We have preliminary evidence that Berenil suppresses LPS-, CpG- and anti-CD40-induced pro-inflammatory cytokine responses by bone marrow-derived macrophages (BMDMs) and dendritic cells (BMDCs) *in vitro*; an effect that is mediated via suppression of MAP kinases and STATs signaling pathways (Kuriakose et al, in preparation). Thus, if Berenil globally alters cytokine production in macrophages, this will also affect IL-10 levels as observed in this study.

Clearance of parasites from the blood of *T. congolense-*infected mice is primarily mediated by macrophages particularly liver (kupffer cells) and splenic macrophages [10]. The uptake of parasites coated with antibodies (including IgM and IgG) results in macrophage activation leading to the production of pro-inflammatory cytokines [2], [5]. In addition, activated macrophages also present trypanosomal antigens and stimulate T cells in an MHC class II-dependent manner to produce IFN-α which further activates macrophages leading to more cytokine production. Thus, massive phagocytosis of trypanosomes (as seen during peak parasitemia) leads to hyper-activation of macrophages and increased production of monokines (IL-1, TNF-α, IL-6, IL-12, monocyte chemotactic protein-1 [MCP-1]) and the T-cell cytokine (IFN-γ). This systemic barrage of cytokine production leads to SIRS and proves fatal to the mouse. We found that in addition to immune hypoactivation, Berenil treatment also significantly lowers serum levels of pro-inflammatory cytokines and suppresses the production of pro-inflammatory cytokines by splenic CD11b^+^ and kupffer cells following LPS stimulation. This suggests that either the macrophage activation machinery or their ability to sense and respond to external stimuli had been altered *in vivo.* Indeed, we found that in uninfected animals, Berenil lowers serum levels of pro-inflammatory cytokines and dampens LPS-induced septic shock following *in vivo* LPS injection. We hypothesize that the dampening of macrophage activation and production of pro-inflammatory cytokines (due probably to direct inhibition of intracellular signaling pathways (MAPK and STATs), reduces the impact of SIRS leading to improved prognostic outcome for the host.

IFN-γ is particularly important in *Trypanosome*-induced cytokine storm. Studies have shown that treatment of BALB/c mice with anti-IFN-γ antibodies prevents acute death in these mice [8]. On the other hand, IFN-γ is necessary for protection because the relatively resistant C57BL/6 mice treated with anti-IFN-γ antibodies become susceptible and die from fulminating parasitemia [28]. Furthermore, IFN-γreceptor deficient C57BL/6 mice succumb acutely to first wave of parasitemia [28]. Interestingly, treatment of the relatively resistant C57BL/6 mice with anti-IL-10R blocking antibody abrogates resistance, which is restored by combined treatment with anti-IFN-γ mAb [29]. These observations suggest that there is a fine balance between the effects of IL-10 and IFN-γ in *T. congolense* infected mice.

In conclusion, we have shown that treatment of *T. congolense*-infected BALB/c mice with Berenil leads to dampening of T cell and macrophage hyper-activation, a lower percentage of FoxP3 regulatory T cells and lowering of systemic pro-inflammatory cytokine levels. These effects were directly associated with dampening of macrophage responses to microbial stimuli *in vitro* and *in vivo*. These findings suggest that in addition to its trypanolytic effects, Berenil also modulates the host immune response, and this may contribute to a more effective parasite control. It is unlikely that the decrease in parasite load resulting from the trypanolytic effect of Berenil is solely responsible for such a significant reduction in pro-inflammatory cytokines and alteration in cellular immune responses given the fact that similar effects were observed in Berenil-treated uninfected mice and following LPS challenge *in vitro* and *in vivo*. Future studies will assess the molecular mechanisms through which Berenil alters cellular responses to microbial stimuli.

## Supporting Information

Figure S1 **Berenil treatment does not affect the frequency of CD4^+^CD25^+^FoxP3^+^ cells in the spleens of uninfected mice.** Splenocytes from naïve (uninfected) BALB/c mice treated or untreated with Berenil were stained directly *ex vivo* with fluorochrome-conjugated mAb against CD4, CD25 and Foxp3 and analyzed by flow cytometry. Presented are representative dot plots showing the expression of CD25^+^ and Foxp3^+^ on CD4^+^ cells. The bar graphs represent the cumulative percentages of CD25^+^ and Foxp3^+^ cells (n = 3 mice per group). The results presented are representative of 2 different experiments with similar results. Bars show mean +/−SEM.
(TIFF)

## References

[1] Magez S, Schwegmann A, Atkinson R, Claes F, Drennan M, et al (2008) The role of B-cells and IgM antibodies in parasitemia, anemia, and VSG switching in Trypanosoma brucei-infected mice. PLoS Pathog 4: e1000122. 18688274 10.1371/journal.ppat.1000122 PMC2483930

[2] Pan W, Ogunremi O, Wei G, Shi M, Tabel H (2006) CR3 (CD11b/CD18) is the major macrophage receptor for IgM antibody-mediated phagocytosis of African trypanosomes: diverse effect on subsequent synthesis of tumor necrosis factor alpha and nitric oxide. Microbes Infect 8: 1209–1218. 16616573 10.1016/j.micinf.2005.11.009

[3] Tomlinson S, Raper J (1998) Natural human immunity to trypanosomes. Parasitol Today 14: 354–359. 17040816 10.1016/s0169-4758(98)01295-2

[4] Taylor KA, Lutje V, Kennedy D, Authie E, Boulange A, et al (1996) Trypanosoma congolense: B-lymphocyte responses differ between trypanotolerant and trypanosusceptible cattle. Exp Parasitol 83: 106–116. 8654538 10.1006/expr.1996.0054

[5] Tabel H, Wei G, Shi M (2008) T cells and immunopathogenesis of experimental African trypanosomiasis. Immunol Rev 225: 128–139. 18837780 10.1111/j.1600-065X.2008.00675.x

[6] PM Kristjanson BM Swallow, GJ Rowlands, RL Kruska, PN de Leeuw (1999) Measuring the costs of Africananimaltrypanosomosis, the potentialbenefits of control and returns to research. Agricultural Systems 59: 79.

[7] Naylor DC (1971) The haematology and histopathology of Trypanosoma congolense infection in cattle. I. Introduction and histopathology. Trop Anim Health Prod 3: 95–100. 5164674 10.1007/BF02356484

[8] Uzonna JE, Kaushik RS, Gordon JR, Tabel H (1998) Experimental murine Trypanosoma congolense infections. I. Administration of anti-IFN-gamma antibodies alters trypanosome-susceptible mice to a resistant-like phenotype. J Immunol 161: 5507–5515. 9820527

[9] Tabel H, Kaushik RS, Uzonna JE (2000) Susceptibility and resistance to Trypanosoma congolense infections. Microbes Infect 2: 1619–1629. 11113381 10.1016/s1286-4579(00)01318-6

[10] Shi M, Wei G, Pan W, Tabel H (2004) Trypanosoma congolense infections: antibody-mediated phagocytosis by Kupffer cells. J Leukoc Biol 76: 399–405. 15136584 10.1189/jlb.1003500

[11] Brack C, Delain E, Riou G, Festy B (1972) Molecular organization of the kinetoplast DNA of Trypanosoma cruzi treated with berenil, a DNA interacting drug. J Ultrastruct Res 39: 568–579. 4556327 10.1016/s0022-5320(72)90122-0

[12] Homeida AM, El Amin EA, Adam SE, Mahmoud MM (1981) Toxicity of diminazene aceturate (Berenil) to camels. J Comp Pathol 91: 355–360. 6274924 10.1016/0021-9975(81)90005-0

[13] Riou G, Benard J (1980) Berenil induces the complete loss of kinetoblast DNA sequences in Trypanosoma equiperdum. Biochem Biophys Res Commun 96: 350–354. 7437039 10.1016/0006-291x(80)91221-8

[14] Gonzalez VM, Perez JM, Alonso C (1997) The berenil ligand directs the DNA binding of the cytotoxic drug Pt-berenil. J Inorg Biochem 68: 283–287. 9397576 10.1016/s0162-0134(97)00111-6

[15] Mahler HR, Perlman PS (1973) Induction of respiration deficient mutants in Saccharomyces cerevisiae by berenil. I. Berenil, a novel, non-intercalating mutagen. Mol Gen Genet 121: 285–294. 4571802 10.1007/BF00433228

[16] Joubert KE, Kettner F, Lobetti RG, Miller DM (2003) The effects of diminazene aceturate on systemic blood pressure in clinically healthy adult dogs. J S Afr Vet Assoc 74: 69–71. 15029949 10.4102/jsava.v74i3.513

[17] Lumsden WH, Herbert WJ, Hardy GJ (1965) In Vivo Prophylactic Activity of Berenil against Trypanosomes in Mice. Vet Rec 77: 147–148. 14330196

[18] Uzonna JE, Kaushik RS, Gordon JR, Tabel H (1999) Cytokines and antibody responses during Trypanosoma congolense infections in two inbred mouse strains that differ in resistance. Parasite Immunol 21: 57–71. 10101716 10.1046/j.1365-3024.1999.00202.x

[19] Rurangirwa FR, Tabel H, Losos GJ, Tizard IR (1979) Suppression of antibody response to Leptospira biflexa and Brucella abortus and recovery from immunosuppression after Berenil treatment. Infect Immun 26: 822–826. 118933 10.1128/iai.26.3.822-826.1979 PMC414692

[20] Otesile EB, Tabel H (1987) Enhanced resistance of highly susceptible Balb/c mice to infection with Trypanosoma congolense after infection and cure. J Parasitol 73: 947–953. 3309242

[21] Tabel H (1982) Activation of the alternative pathway of bovine complement by Trypanosoma congolense. Parasite Immunol 4: 329–335. 7145463 10.1111/j.1365-3024.1982.tb00444.x

[22] Lanham SM, Godfrey DG (1970) Isolation of salivarian trypanosomes from man and other mammals using DEAE-cellulose. Exp Parasitol 28: 521–534. 4993889 10.1016/0014-4894(70)90120-7

[23] Wei G, Tabel H (2008) Regulatory T cells prevent control of experimental African trypanosomiasis. J Immunol 180: 2514–2521. 18250461 10.4049/jimmunol.180.4.2514

[24] Guilliams M, Oldenhove G, Noel W, Herin M, Brys L, et al (2007) African trypanosomiasis: naturally occurring regulatory T cells favor trypanotolerance by limiting pathology associated with sustained type 1 inflammation. J Immunol 179: 2748–2757. 17709488 10.4049/jimmunol.179.5.2748

[25] Okwor I, Onyilagha C, Kuriakose S, Mou Z, Jia P, et al (2012) Regulatory T cells enhance susceptibility to experimental trypanosoma congolense infection independent of mouse genetic background. PLoS Negl Trop Dis 6: e1761. 22860150 10.1371/journal.pntd.0001761 PMC3409116

[26] Ding L, Linsley PS, Huang LY, Germain RN, Shevach EM (1993) IL-10 inhibits macrophage costimulatory activity by selectively inhibiting the up-regulation of B7 expression. J Immunol 151: 1224–1234. 7687627

[27] Kaushik RS, Uzonna JE, Zhang Y, Gordon JR, Tabel H (2000) Innate resistance to experimental African trypanosomiasis: differences in cytokine (TNF-alpha, IL-6, IL-10 and IL-12) production by bone marrow-derived macrophages from resistant and susceptible mice. Cytokine 12: 1024–1034. 10880248 10.1006/cyto.2000.0685

[28] Magez S, Radwanska M, Drennan M, Fick L, Baral TN, et al (2006) Interferon-gamma and nitric oxide in combination with antibodies are key protective host immune factors during trypanosoma congolense Tc13 Infections. J Infect Dis 193: 1575–1583. 16652287 10.1086/503808

[29] Shi M, Pan W, Tabel H (2003) Experimental African trypanosomiasis: IFN-gamma mediates early mortality. Eur J Immunol 33: 108–118. 12594839 10.1002/immu.200390013

