# Supplementary figures and images for: Diminazene Aceturate (Berenil) Modulates the Host Cellular and Inflammatory Responses to Trypanosoma congolense Infection
Source: PLoS One. 2012 Nov 7;7(11):e48696. doi: 10.1371/journal.pone.0048696 (PMC3492428; doi:10.1371/journal.pone.0048696)

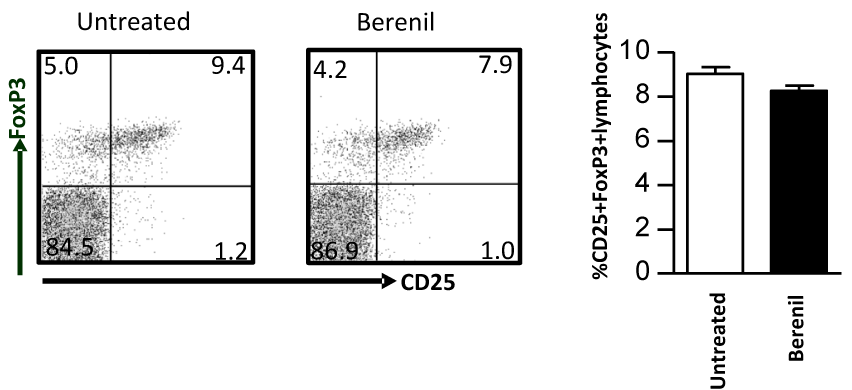

Supplement: Figure S1 — Berenil treatment does not affect the frequency of CD4+CD25+FoxP3+ cells in the spleens of uninfected mice. Splenocytes from naïve (uninfected) BALB/c mice treated or untreated with Berenil were stained directly ex vivo with fluorochrome-conjugated mAb against CD4, CD25 and Foxp3 and analyzed by flow cytometry. Presented are representative dot plots showing the expression of CD25+ and Foxp3+ on CD4+ cells. The bar graphs represent the cumulative percentages of CD25+ and Foxp3+ cells (n = 3 mice per group). The results presented are representative of 2 different experiments with similar results. Bars show mean +/−SEM. (TIFF) [file pone.0048696.s001.tiff]
